# Assessment of Knowledge About Human Papillomavirus Vaccination Among Primary School Girls in Arba Minch Town, South Ethiopia, 2020 an Institution-Based Cross-Sectional Study

**DOI:** 10.2147/CMAR.S359413

**Published:** 2022-07-18

**Authors:** Eshetu Yisihak Ukumo, Feleke Gebremeskel Woldehawariat, Samuel Abebe Dessalegn, Desta Markos Minamo, Gebresilasea Gendisha Ukke

**Affiliations:** 1 Department of Midwifery, College of Medicine and Health Sciences, Arba Minch University, Arba Minch, Ethiopia; 2 School of Public Health, College of Medicine and Health Sciences, Arba Minch University, Arba Minch, Ethiopia

**Keywords:** cervical cancer, human papillomavirus

## Abstract

**Purpose:**

This study aimed to determine the knowledge of human papillomavirus vaccination (PHV) and associated factors among primary school girls in Arba Minch town, South Ethiopia, in 2020. Hence, the levels of knowledge towards the HPV vaccination of girls were assessed and recommended to the concerned bodies. Clinically, this study could increase the acceptance of HPV vaccination after the intervention of the concerned bodies to improve adolescents’ knowledge levels. As a result, it could decrease the incidence of cervical cancer. Socially, this study may increase the research involvement of adolescents, communities, and stakeholder groups.

**Patients and Methods:**

A school-based cross-sectional study involving 537 female students was conducted on January 24, 2020. The study participants were selected by a simple random sampling technique. A pre-tested questionnaire was used to collect the data. The logistic regression model was used to identify the statistically significant variables for knowledge of the human papillomavirus vaccination.

**Results:**

The overall knowledge level of the participants in this study was 71.7%. Their main source of information is social media (41.74%), followed by medical staff (29.69%). Age ≥15 years old, AOR =3.74, 95% CI (2.20_6.37), P-value <0.001, grade level of 7 and 8, AOR =3.98, 95% CI (2.40_6.58), P-value <0.001, mother’s educational status of secondary and more than secondary, and father’s educational status of more than secondary, AOR=13.60, 95% CI (5.69_32.53) P-value <0.001, 22.27, 95% CI (8.23_60.30), P-value<0.001, 2.18, 95% CI (1.09_4.35), P-value 0.03, respectively, and access to HPV vaccination information (AOR = 8.65, 95% CI (3.92_19.07), P-value 0.001) were associated with overall knowledge.

**Conclusion:**

Nearly three-fourths of the study participants were knowledgeable about human papillomavirus vaccination. Knowledge about the human papillomavirus vaccination shows a positive association with age, education level, parents’ educational status, and access to information sources.

## Introduction

The HPV vaccine protects against infection with the human papillomavirus (HPV). The human papillomavirus (HPV) is a virus that causes over 200 diseases, 40 of which are spread through sexual contact. More than a dozen HPV strains can cause cancer in the anal, cervical, vulvar, oropharyngeal, penile, vulvar, and vaginal locations, whereas two HPV variants can cause genital warts.^1^ Cervical cancer is a worldwide public health issue, with 528, 000 new cases and 266, 000 deaths per year. Nearly 70% of the global load is borne by countries with lower levels of development.^2^ Cervical cancer is the most notable health problem in Sub-Saharan Africa (SSA). Annually, 34.8/100,000 women’s new cases and 22.5/100,000 women’s deaths occurred in this sub-region.^2^ In Ethiopia, cervical cancer is the second leading cause of cancer mortality. Every year, 7095 women are diagnosed and 4732 die from cervical cancer.^2–5^ Of the total served patients at the center, women made up about 73%, and cervical cancer is the most common disease, comprising over one-third of all female patients treated in Ethiopia.^6^

The majority of cancers (over 80%) in sub-Saharan Africa (SSA) are detected at a late stage, predominantly due to a lack of information about cervical cancer and a scarcity of prevention services, which in turn are associated with low survival rates after surgery or radiotherapy.^3^ The primary prevention strategy for cervical cancer is to avoid the transmission of HPV types. Among those methods, HPV vaccination is one.^3^ Similarly, with WHO, Ethiopia recommends the primary target population of girls aged 9–14 years old for HPV vaccination before becoming sexually active.

However, in many countries, knowledge and acceptance of HPV vaccination are low. For example, knowledge in China was 15.7%,^7^ Taiwan was 7.49%,^8^ Spain was 67%,^9^ Grace was 59.1%,^10^ Turkey was 23.2%,^11^ Nigeria was 23%,^12^ Serbia was 14.2%,^13^ Ghana was 40%,^14^ Ethiopia (Addis Ababa was 13%),^15^ and Jimma was 43.8%,^16^ and acceptance of HPV vaccination in Hong Kong of China was 36.5%,^17^ the Netherlands was 39%,^18^ Turkey was 11.2%,^19^ and Senegal was 28%.^20^

Factors that are associated with the knowledge of HPV vaccination include information source,^14^ residences,^21^ family education levels,^22^ ages,^16^ and school type, discussion with health care providers, and^22^ school-based education about HPV vaccination.^7^

Even if knowledge of HPV vaccination affects HPV vaccination uptake among adolescent girls, knowledge of HPV vaccination was limited in Ethiopia. Therefore, this study aimed to fill this information gap by assessing knowledge and associated factors of HPV vaccination among primary school girls in Arba Minch town, south Ethiopia. The primary beneficiaries of this study were adolescent girls because their levels of knowledge regarding the HPV vaccination were assessed and recommended to the concerned bodies. Clinically, this study could increase the acceptance of HPV vaccination after the intervention of the concerned bodies, such as developing age-and gender-based health education to improve adolescents’ knowledge levels based on the recommendations. As a result, it could decrease the incidence of cervical cancer, which is the second most common cancer in Ethiopia. Socially, this study may increase the research involvement of adolescents and communities as well as invite stakeholder groups such as educational institutions, health institutions, health policymakers, and nongovernmental organizations to participate in relevant interventions. The results of this study could also be a crucial source of information for health program planners and further investigators. This can benefit already existing literatures by bringing updated information.

## Materials and Methods

### Study Setting

Arba Minch town, the capital city of Gamo zone, is 505 km from Addis Ababa (the capital city of Ethiopia) and about 280 km from Hawasa (the capital city of the southern nationalities and peoples region of Ethiopia). There are two hospitals, two health centers, and 11 health posts providing health services. Arba Minch town has four sub-cities and eleven kebeles (the lowest administrative unit in Ethiopia). Arba Minch town has 55 kindergarten (KG) schools, where schoolchildren attend from 4 to 6 years of age before starting grade one, of which 6 are public. There are also 18 full primary schools (grade one up to eight), of which eight are public, and nine secondary and preparatory schools (grade nine up to twelve), of which five are public. The total number of students, without adding kindergarten students, registered in 2020 in Arba Minch town was 27,512, of which 14,431 were female students. For our study purposes, we used primary school students who were in grades five through eight, with a total number of 8867, of which 4758 were female students. The age distribution of female students in this grade level during our study period was 12 to 17 (ArbaMich City Education Office, Personal Communication, December, 2020). Zonal and city health offices work jointly with schools to deliver the vaccination in a school-based way after creating awareness through health extension workers and other concerned bodies (ArbaMich City and Zonal Health Office, Personal communication, December 2020). Students were classified as rural or urban based on their parents’ habitations. For those students who are classified as rural, their parents live in a rural area very close to Arba-Minch town, and the students come to city only for educational purposes; they return to their parents for habitation. Some others dwell in the city with their guardians for working days only.

### Study Design

A school-based cross-sectional study

### Source Population

All girl students in primary schools of Arba Minch town, South Ethiopia

### Study Population

All girl students in 6 selected primary schools of Arba Minch town during the data collection period.

### Study Variables

#### Dependent Variable

Knowledge of HPV vaccination.

#### Independent Variables

Age, Grade level, School type, Educational status of family, Source of information about HPV vaccination, School based education about HPV vaccination,

### Operational and Term Definitions

Good knowledge: those who answered 50% or more or 4 and above from 7 knowledge assessing questions.^16^

Poor knowledge: those who answered below (50%) from knowledge assessing questions.^16^

The human papillomavirus: is a group of viruses that is extremely common worldwide.^23^

The human papillomavirus vaccine: is a vaccine used to protects against infection with the human papillomavirus (HPV).^1^

### Eligibility Criteria

#### Inclusion Criteria

Those girls who were attending a primary school and in grade 5 up to 8.

#### Exclusion Criteria

Those girls who were ill at the time of data collection.

### Sample Size Determination

The sample size was determined by using a single population proportion formula with the assumption of a 95% confidence level, 5% margin of error, by taking “p” (0.696) from similar studies conducted among adolescent girls,^24^ design effect 1.5 and adding 10% non-response rate. The final sample size was 537.

### Sampling Technique

From a total of 18 primary schools found in the city (both private and public), 6 primary schools were selected by simple random sampling without replacement through the lottery method. Then the calculated sample size was allocated to each school proportionally. After getting eligible adolescent girls for each school from their homeroom teachers, we relisted to prepare a sampling frame for that school. While preparing the sampling frame, a section of given students was listed in front of their names to finally get them, and then study units of a given school were obtained by a simple random sampling technique. Finally, selected study units for given schools were requested to be in one place (to one of the sections at break time) and fill in the questionnaire through the guidance of data collection facilitators and supervisors.

### Data Collection Tool

A pre-tested, semi-structured, translated into Amharic language, and self-administered questionnaire was used (Annex: 3). The questionnaire was developed after an extensive review of published articles. Data collectors were selected from among the teachers of their respective schools. The questionnaire consists of socio-demographic information and knowledge of the HPV vaccination among adolescent girls of Arba Minch town. The tool was prepared with the English version and translated to Amharic to avoid missing important information; then it was translated back to English for analysis.

### Data Quality Control

The training was given on the clarification of some assessment tools, the aim of the study, time of data collection, timely collection and reorganization of the collected data from respective schools, and submission on due time. The questionnaire was pre-tested with 5% of the sample size in Mirab Abaya primary school in the Gamo zone, which is not included in this study. In addition to appropriate recruitment and training of data collectors, the quality of the data was monitored frequently both in the field and during data entry. This was done in the field through close supervision of data collectors. All filled questionnaires were examined for completeness and consistency during data collection.

### Data Processing and Analysis

Data was entered to Epi data version 4.6 and exported for analysis to SPSS Version 23. Descriptive analysis was made and measures of central tendency were also determined. In scoring knowledge, one point was awarded for every correct answer and zero for incorrect or “do not know” responses. The total knowledge score was then be converted into percentages and the level of knowledge of the respondents was classified based on their score and of overall 7 knowledge questions, study participants who score <50% (ie answered correctly ≤3 questions) were labeled as having poor knowledge and those who answer 4 or above from 7 questions or score 50% and above were labeled as having good knowledge.

Logistic regression was applied to see the association between dependent and independent variables. Independent variables, which had an association in bi-variable analysis with a p-value <0.25 were entered into the multivariable logistic regression model. Independent variables with a p-value<0.05 in the multivariable logistic regression model were considered as statistically significant factors for knowledge. The results were presented as odds ratios (OR) with 95% confidence intervals. An odds ratio with a corresponding 95% CI was used to quantify the association between a dependent variable and independent variables. The model fitness was checked by using the software application of the Hossmare and Lame show test (>0.05). The results were presented using text, and tables.

## Results

Out of the 537 samples that were distributed, 516 study participants were interviewed in this study, with a response rate of 96.1%. The majority of study participants (71.3%) were Gamo in ethnicity and protestant Christians in religion (60.5%) (Table 1) Gamo is an ancient people speaking an Omotic language in Ethiopia whose administrative territory is Gamo Zone with a capital city of Arba Minch. Zone in Ethiopia is the third administrative unit that is Kebele, Woreda/district /, Zone, region and finally federal administrative unit, which is the highest administrative unit in Ethiopia.

**Table 1:** Socio-Demographic Characteristics of Primary School Girls in Arba Minch Town, Southern Ethiopia, 2020, (N=516)

Variables	Categories	Frequency (n)	Percent (%)
Age in years	12_14	284	55
	≥15	232	45
Religion	Protestant	312	60.5
	Orthodox	148	28.7
	Catholic	20	3.9
	Muslim	17	3.3
	Others	19	3.7
Ethnic group	Gamo	368	71.3
	Gofa	46	8.9
	Amhara	33	6.4
	Oromo	23	4.5
	Wolayta	33	6.4
	Others	13	2.5
Residence	Urban	436	84.5
	Rural	80	15.5
Grade level	Grade 5and 6	219	42.4
	Grade 7 and 8	297	57.6
School type	Public	470	91.1
	Private	46	8.9
Mothers educational status	No education	112	21.7
	Primary education	115	22.3
	Secondary education	184	35.7
	More than secondary	105	20.3
Fathers educational status	No education	72	14.0
	Primary education	89	17.2
	Secondary education	107	20.7
	More than secondary	248	48.1

### Knowledge of Respondents Toward Human Papilloma Virus (HPV) Vaccination

Three hundred seventy-one, 71.7% CI, 95% (67.6_75.4) of the study participants were found to have good knowledge about the HPV vaccination. 69.8% of the study participants knew the targeted age group and 78.3% knew the number of HPV vaccinations to be given for the prevention of cervical cancer. The main source of information was social media (41.74%), followed by health care providers while giving health information at school (29.69%) (Table 2)

**Table 2:** Knowledge and Related Responses of Primary School Girls in Arba Minch Town, South Ethiopia, 2020, (N=516)

Variables		Frequency	Percent
Do you know HPV vaccination	Yes	409	79.3
	No	107	20.7
What is HPV vaccination	Given for prevention of cervical cancer	310	60.1
	Given for prevention of malaria	50	9.7
	Given for prevention of HIV ADDIS	16	3.1
	I do not know	140	27.1
An interval that someone take second HPV vaccination	6 month	314	60.9
	6 year	62	12.0
	1 month	26	5.0
	3 month	114	22.1
HPV vaccination should be taken before sex initiations	Yes	292	56.6
	No	224	43.4
Number of HPV vaccination	Twice	404	78.3
	Once	112	21.7
Possible to have cervical cancer screening after HPV vaccination	Yes	383	74.2
	No	133	25.8
Age at which someone undergo HPV vaccination	9–14 years	360	69.8
	18–21 years	30	5.8
	All ages	27	5.2
	I do not know	99	19.2
Obtained information about HPV vaccination from any source	Yes	357	69.2
	No	159	30.8
Information source	Social media	149	41.74
	Health care workers	106	29.69
	Families and friends	102	28.57
In your school, is there any reproductive health related clubs	Yes	357	69.2
	No	159	30.8
Knowledge level	Poor Knowledge	146	28.3 CI 95%(24.6_32.4)
	Good Knowledge	370	71.7 CI 95%(67.6_75.4)

**Abbreviations**: HPV, human papilloma virus; CI, confidence interval; HIV, human immunodeficiency virus; AIDs, acquired immunodeficiency syndrome.

### Associated Factors with Knowledge of Human Papilloma Virus (HPV) Vaccination

Logistic regression analysis was done to assess an association between the socio-demographic characteristics of the study participants with their knowledge level towards HPV vaccination. It showed that; age, grade level, familial educational status, and information source had a positive association. That is, those who are ≥15 years old have a close to fourfold greater likelihood of having good knowledge about HPV vaccination as compared to those who are 12–14 years old. AOR =3.74, 95% CI (2.20_6.37), P-value (<0.001),

Those who are in Grade 7 and 8 are close to four-fold more likely to have good knowledge about HPV vaccination as compared to those who are in Grade 5 and 6. AOR = 3.98, 95% CI (2.40_6.58), P-value (<0.001).

Familial educational status: those whose mothers’ educational status is secondary close to fourteen fold and whose fathers’ educational status is more than secondary close to threefold have better knowledge about HPV vaccination than their counterparts. AOR=13.60, 95% CI (5.69_32.53) P-value <0.001), 22.27, 95% CI (8.23_60.30), P-value<0.001), 2.18, 95% CI (1.09_4.35), P-value 0.03) respectively,

Those who obtained HPV vaccination information have a close to nine-fold higher likelihood of having good knowledge about HPV vaccination than those who do not obtain HPV vaccination information. AOR=8.65, 95% CI (3.92_19.07), P-value (<0.001) (Table 3). Hossmer and Leme show’s test value of this model was 0.131.

**Table 3:** Association Between Socio-Demographic Factors and Knowledge Level of HPV Vaccination

Variables		Knowledge of HPV Vaccine		COR(95% CI)	AOR(95% CI)	P _value
		Good	Poor			
Age	12_14	180(34.9%)	104(20.2%)	1	1	1
	≥15	190(36.8%)	42(8.2%)	2.61(1.73_3.95)	3.74(2.20_6.37)	<0.001** *
School type	Public	330(64%)	140(27.1%)	1	1	1
	Private	40(7.8%)	6(1.2%)	2.83(1.17_6.82)	1.704(0.6_4.87)	0.32
Residence	Rural	52(10.1%)	28(5.4%)	1	1	1
	Urban	318(61.6%)	118(22.9%)	1.45(0.88_2.41)	1.27(0.68_2.39)	0.46
Grade level	5 and 6	136(26.4%)	83(16.1%)	1	1	1
	7and 8	234(45.3%)	63(12.2%)	2.27(1.54_3.35)	3.98(2.40_6.58)	<0.001***
Mothers’ educational status	No education	64(12.4%)	48(9.3%)	1	1	1
	Primary	58(11.2%)	57(11%)	0.76(0.45_1.29)	0.94(0.50_1.76)	0.84
	Secondary	155(30%)	29(5.6%)	4.01(2.32_6.92)	13.60(5.69_32.53)	<0.001***
	More than secondary	93(18%)	12(2.3%)	5.81(2.86_11.80)	22.27(8.23_60.30)	<0.001***
Fathers’ educational status	No education	37(7.2%)	35(6.8%)	1	1	1
	Primary	46(8.9%)	43(8.3%)	1.01(0.54_1.88)	0.73(0.35_1.55)	0.41
	Secondary	83(16.1%)	24(4.7%)	3.27(1.71_6.25)	1.98(0.90_4.36)	0.09
	More than secondary	204(39.5%)	44(8.5%)	4.39(2.49_7.72)	2.18(1.09_4.35)	0.03*
Herd about HPV vaccination from any source	Yes	260(50.4%)	97(18.8%)	1	1	1
	No	110(21.3%)	49(9.5%)	1.19(0.79_1.80)	8.65(3.92_19.07)	<0.001***
Reproductive health related information in school	Yes	254(49.2%)	103(20%)	0.91(0.60_1.39)	0.93(0.55_1.57)	0.77
	No	116(22.5%)	43(8.3%)	1	1	1

**Notes**: *p<0.05: statistically significant ***p<0.001: strongly statistically significant 1: Reference category.

**Abbreviations**: HPV, human papilloma virus; COR, crude odd ratio; AOR, adjusted odd ratio; CI, confidence interval.

## Discussion

This study’s overall knowledge score was 71.7% CI 95% (67.6_75.4), which is lower than studies conducted in Australia (97.4%),^16^ Zambia 98.7%,^17^ and higher than the United States 47.5%,^18^ china 15.7%,^12^ Taiwan7.49%,^19^ Spain 67%,^11^ Grace 59.1%,^20^ and Turkey 23.2%,^21^ Nigeria 23%,^22^ Serbia 14.2%, Ghana 40%,^13^ Minakulu sub-county in northern Uganda 69.6%,^24^ and Ethiopia (AddisAbaba13%, and Jimma 43.8%.^10^ These dissimilarities may be due to the differences in culture and information access, in educational institutions’ structures, sampling procedures, tools, and the cut-off points used to define the level of knowledge.

The age of respondents had a positive association with knowledge. Those who are >15 years old are close to fourfold more likely to have good knowledge about HPV vaccination as compared to those who are 12_14 years old. AOR =3.74, 95% CI (2.20_6.37), P-value (<0.001), which is similar to a study conducted in Greek^20^ and Jimma, Ethiopia,^10^ the association might be due to the fact that as their age increases, they might be allowed to take part in different activities like reproductive health clubs, sex education, and following social media. This might have helped them to get additional information, including information about HPV vaccination.

As the education level of girls’ increases, so does knowledge of the HPV vaccination. Those who are in Grade 7 and 8 are close to four-fold more likely having good knowledge about HPV vaccination as compared to those who are in Grade 5 and 6. AOR=3.98, 95% CI (2.40_6.58), P-value <0.001). A similar finding was found in a study conducted in Jimma, southwest Ethiopia,^10^ and Turkey.^21^ The association might be due to the increased level of education that may pave the way to increased information access, and understanding.

Familial educational status (those for whom mothers’ educational status of secondary close to fourteen fold and more than twenty-two fold and fathers’ educational status of more than secondary close to twofold have good knowledge about HPV vaccination than their counterparts). AOR=13.60, 95% CI (5.69_32.53) P-value (<0.001), 22.27, 95% CI (8.23_60.30), P-value (<0.001) 2.18, 95% CI (1.09_4.35) P-value (0.03) respectively. This is similar to a study conducted in Senegal.^23^ It could be because of good communication between parents and their daughters, which may have made them aware.

Information sources about HPV vaccination have a statistically significant association with knowledge of HPV vaccination. That is, those who obtain HPV vaccination information have a close to nine-fold higher likelihood of having good knowledge about HPV vaccination than those who do not obtain HPV vaccination information. AOR = 8.65, 95% CI (3.92–19.07), P-value <0.001, which is similar to a study conducted in the USA.^18^ The association might be due to taking part in different activities like reproductive health clubs, sex education, following social media and information access. This might have helped them to get information about the HPV vaccination.

This is significant both clinically and socially. Clinically, after the involvement of the relevant bodies, such as providing age-and gender-based health education to raise adolescents’ knowledge levels based on the guidelines, this study may increase the acceptance of the HPV vaccine. As a result, cervical cancer, Ethiopia’s second-most frequent cancer, could become less common. In terms of social implications, this study may boost adolescent and community participation in research as well as invite stakeholder groups like educational institutions, health institutions, health officials, and nongovernmental organizations to participate in relevant interventions. The findings of this study could be useful to health-care planners and researchers in the future. This can help to update existing publications.

## Strength of the Study

The quality of the study was unquestionable as it was conducted in a multidisciplinary way. In addition to this, a pretest was conducted and the relevance, consistency, and validity of the tool were checked. It was able to survey a random sample of female students, thereby increasing the generalizability of the findings to other eligible students. This study utilized a self-administered questionnaire, which increases the likelihood of respondents answering openly, hence increasing internal validity. This study provides new and updated information about adolescents’ knowledge of HPV vaccination, which can serve as a baseline for future researchers as well as health policymakers and implementers.

## Limitations of the Study

The limitation of this study was that it did not include the girls’ parents in the study as they were decision-makers. Parents’ knowledge and attitude can influence their acceptance of HPV vaccination for their daughters, thereby affecting their actual utilization of HPV vaccination. In our country, adolescents less than 18 years old cannot decide by themselves but through their parents or guardians. Therefore, the researcher/s should consider the parents of the adolescents to cover their attitudes, acceptance, and knowledge of HPV vaccination. This study also did not cover the attitudes of the adolescents as well as the communities, which are the important factors to consider. Our study included only a few rural students due to different resource and technical problems. The rural areas are the places where there is limited access to information, so the next researcher should consider it.

## Conclusions

The overall knowledge level of study participants was 71.7%, which is still less than three-quarters. Factors positively associated with knowledge of HPV vaccination were age, grade level, and familial educational status, as well as information source. We recommend that health institutions prepare age-and gender-based health education to further improve adolescent knowledge and those educational institutions encourage female education as well as participation in health and health-related clubs. Researcher who delighted in investigating the same thematic area better to incorporate parents or guardians.

## Data Sharing Statement

The data supporting the conclusions of this study were included in the text.
